# Particle-Size Variability of Aerosol Iron and Impact on Iron Solubility and Dry Deposition Fluxes to the Arctic Ocean

**DOI:** 10.1038/s41598-019-52468-z

**Published:** 2019-11-13

**Authors:** Yuan Gao, Christopher M. Marsay, Shun Yu, Songyun Fan, Pami Mukherjee, Clifton S. Buck, William M. Landing

**Affiliations:** 1 Department of Earth and Environmental Science, Rutgers University, Newark, NJ 07102 USA; 2 Skidaway Institute of Oceanography University of Georgia, Savannah, GA 31411 USA; 3 Department of Earth, Ocean, and Atmospheric Science, Florida State University, Tallahassee, FL 32306 USA

**Keywords:** Biogeochemistry, Environmental sciences, Ocean sciences

## Abstract

This study provides unique insights into the properties of iron (Fe) in the marine atmosphere over the late summertime Arctic Ocean. Atmospheric deposition of aerosols can deliver Fe, a limiting micronutrient, to the remote ocean. Aerosol particle size influences aerosol Fe fractional solubility and air-to-sea deposition rate. Size-segregated aerosols were collected during the 2015 US GEOTRACES cruise in the Arctic Ocean. Results show that aerosol Fe had a single-mode size distribution, peaking at 4.4 µm in diameter, suggesting regional dust sources of Fe around the Arctic Ocean. Estimated dry deposition rates of aerosol Fe decreased from 6.1 µmol m^−2^ yr^−1^ in the areas of ~56°N–80°N to 0.73 µmol m^−2^ yr^−1^ in the areas north of 80°N. Aerosol Fe solubility was higher in fine particles (<1 µm) which were observed mainly in the region north of 80°N and coincided with relatively high concentrations of certain organic aerosols, suggesting interactions between aerosol Fe and organic ligands in the high-latitude Arctic atmosphere. The average molar ratio of Fe to titanium (Ti) was 2.4, substantially lower than the typical crustal ratio of 10. We speculate that dust sources around the Arctic Ocean may have been altered because of climate warming.

## Introduction

Iron (Fe) is a limiting micronutrient for phytoplankton growth in surface waters of the remote ocean as demonstrated by meso scale Fe addition experiments^1–3^. As such, Fe availability has the potential to affect the global cycles of other elements of climatic importance such as nitrogen^4^, both at present and throughout geologic time^5^. The major natural source of Fe to remote ocean surface waters is aeolian dust transported from arid continental regions^6–9^. Atmospheric Fe also comes to the ocean from other sources, such as combustion^10,11^, biomass burning^12^ and volcanic emissions^13,14^. The Fe supply has been found to limit primary production in certain subarctic regions^15,16^. Fe scarcity, along with nitrogen and light, could also control primary productivity in areas of the Arctic Ocean^17^.

The availability of Fe for oceanic phytoplankton uptake, known as Fe bioavailability, depends on the Fe solubility in aerosol particles, and this important property is partially a function of the size distribution of Fe-containing particles^18,19^. As a transition metal, Fe in aerosols can also be involved in chemical reactions with other atmospheric species, such as the oxidation of S(IV) to S(VI) in cloud water^20^, contributing to the oxidation capacity of the atmosphere. The Fe reaction efficiency may depend on the surface area and size distributions of Fe-containing aerosol particles as well as Fe speciation^21,22^. Thus, quantifying the size distribution of Fe-containing particles and aerosol Fe fractional solubility is critically important in global biogeochemical cycles and climate studies.

The Arctic Ocean has experienced rapid alteration in recent decades including significant reductions in sea ice extent and thickness as well as enhanced runoff from the surrounding continents in response to climate warming^23–26^. Atmospheric transport and deposition of aerosols is an important mechanism for Fe and other trace elements from natural and anthropogenic sources from the surrounding continents to enter the Arctic Ocean^27–31^. While many studies on aerosols over the Arctic have focused on the winter and springtime Arctic haze, a phenomenon resulting from efficient meridional transport and low rates of wet deposition^32–36^, fewer studies of aerosols have been conducted over the central Arctic Ocean in summer and fall^37,38^. At these times, the polar front retreats northwards, reducing the transport of polluted air from mid-latitude sources^32^, and this time period therefore provides a unique window to explore the background composition of the marine atmosphere over the Arctic.

The goal of this study is to characterize the particle size distribution of aerosol Fe and its fractional solubility measured by Fe speciation in the late summer/early autumn Arctic marine atmosphere. Size-segregated aerosol particles were sampled using a Micro-Orifice Uniform Deposition Impactor (MOUDI) during the US GEOTRACES Western Arctic (GN01) cruise along a transect of 56°N–90°N in August–October 2015. We investigated the spatial variability of aerosol Fe particle size and aerosol Fe solubility based on the concentrations of aerosol Fe and its speciation in the particulate phase. We also explored the partitioning of Fe between fine- and coarse-mode particles. The new aerosol Fe size-distribution data enabled us to generate spatially variable dry deposition rates in order to calculate the atmospheric dry deposition of Fe to the Arctic basin, which is important for evaluating the relative significance of dry vs wet deposition of Fe. The measurements provide insight into the potential climate induced changes in the Arctic environment.

## Results

### Spatial variations of total aerosol Fe

**Figure 1:**
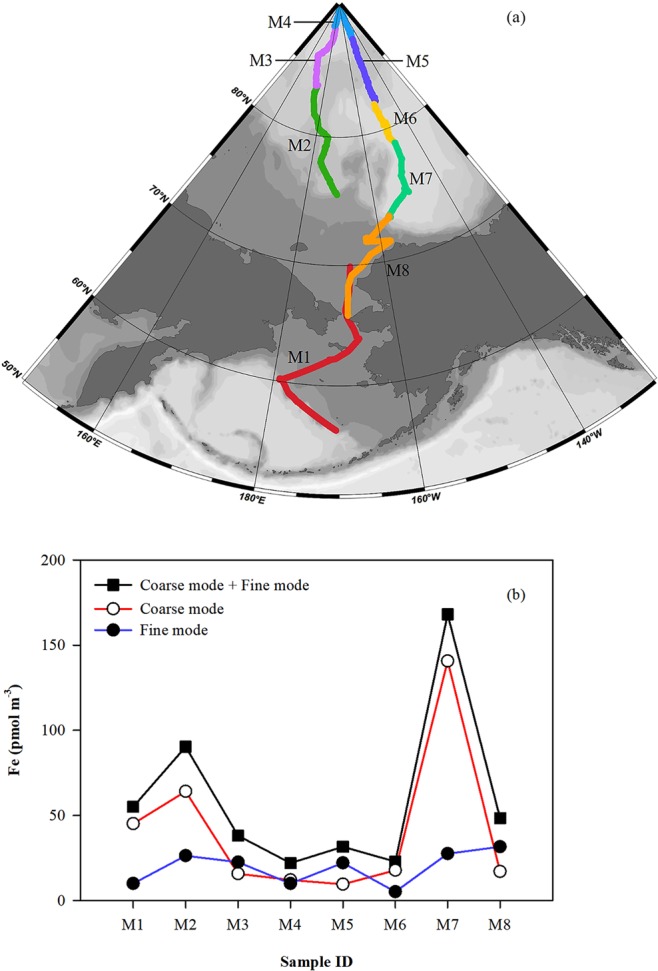
(**a**) Study region (generated using Matlab R2016b (9.1.0.441655), https://www.mathworks.com/products/matlab.html). Cruise track shows the coverage of eight sets of size-segregated aerosol samples collected by a ten stage Micro-Orifice Uniform Deposition Impactor (MOUDI; MSP Corporation, MN, USA) (M1-M8). Air sampling on this cruise started in the Bering Sea with M1, continued toward the North Pole, and returned south, finishing with M8. (**b**) Spatial variations of aerosol Fe in fine- and coarse-mode particles and total (coarse + fine) aerosol Fe derived from eight sets of samples.

Eight MOUDI aerosol samples were collected along a transect between 56°N and 90°N (Fig. 1a). The concentration of total aerosol Fe for each MOUDI sample (∑Fe_T_) was defined as the sum of strong acid-digestible aerosol Fe (Fe_T_) measured on each of ten MOUDI stages, with values of ∑Fe_T_ ranging from 22.0 pmol m^−3^ to 168 pmol m^−3^ and an average (±one standard deviation) of 59.5 (±49.1) pmol m^−3^ (Fig. 1b). These concentrations were substantially lower than bulk aerosol Fe values reported by others during winter and spring^39^, when both natural dust and anthropogenic sources contributed. The highest ∑Fe_T_ loading was found in sample M7 (168 pmol m^−3^) collected between 73°N–79°N and 148°W–157°W. Based on air mass back-trajectory analyses (AMBT), this sample was affected by air masses that were characterized by mixtures of polar and continental air. The second highest concentration of ∑Fe_T_ occurred in sample M2, collected in the area of 75°N–83°N, 171°W–175°E. Although M2 was largely affected by polar air, extended AMBT analyses (up to 10 days) suggested that this sample might be affected by the residuals of upper-level transport of air from the coastal region of the Laptev Sea. Possibly, local dust sources in Siberia and Alaska could contribute to the observed aerosol Fe^40^.

In this study, particles were partitioned between the coarse and fine mode at an aerodynamic diameter of 1 µm. Aerosol Fe in coarse particles showed a similar distribution pattern as that of ∑Fe_T_, ranging from 9.53 pmol m^−3^ to 141 pmol m^−3^ (Fig. 1b). Relatively high concentrations of coarse-mode (>1 µm) aerosol Fe were found in samples M1, M2, and M7, which were largely collected in areas south of 80°N. Aerosol Fe in samples collected north of 80° (M3, M4, M5 and M6) was more evenly distributed between the coarse and fine modes. Sample M8 is an exception to this, having relatively low amounts of coarse aerosol Fe despite being collected between 73°N–66°N. Excluding M8, the observed spatial distribution of Fe in coarse particles could be attributed to the preferential removal of larger particles during atmospheric transport in the marine atmospheric boundary layer over the Arctic Ocean. Fine-mode aerosol Fe concentrations were relatively low throughout the study, ranging from 5.14 pmol m^−3^ to 31.5 pmol m^−3^, and did not exhibit large fluctuations among samples (or with latitude), suggesting a relatively uniform distribution and long atmospheric residence time of Fe in fine particles over this region.

Calculations of crustal enrichment factors (EF) relative to titanium (Ti), as a first step of source identification^41^, showed that the average EF values from these samples were 0.35 (±0.28) for coarse particles and 0.23 (±0.20) for fine particles. These results indicate that aerosol Fe was not enriched relative to average upper continental crust, and EFs of <1 may suggest a certain degree of Fe depletion in the source materials, relative to crustal averages. Similarly, the variability in EF could reflect natural variability in the composition of source rocks. Similar low Fe/Ti ratios have been observed in remote aerosols in other studies^42–44^ and in bulk aerosols collected during the same research cruise^38^.

### Particle-size distributions of aerosol Fe in the Arctic marine atmosphere

**Figure 2:**
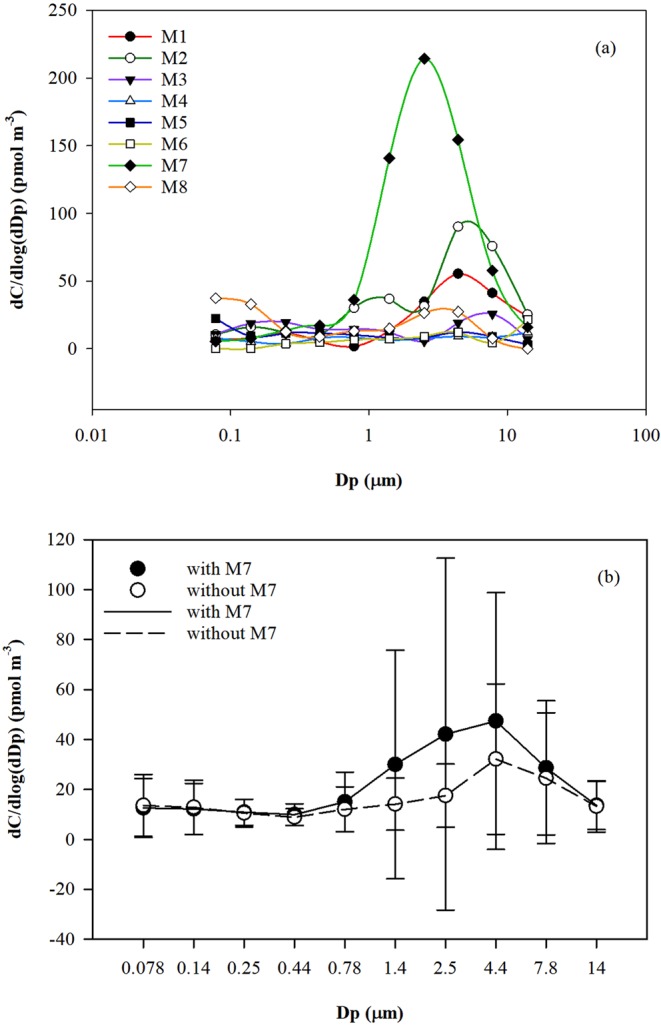
(**a**) Mass size distributions of aerosol Fe in eight sets of MOUDI samples. (**b**) A combined particle size distribution of aerosol Fe (pmol m^−3^): solid circles and line show the average concentrations of Fe in each sampling stage across all MOUDI deployments, and open circles and dashed line represent the same, with M7 excluded. Vertical bars represent the standard deviation of the Fe concentration on individual stages.

Aerosol Fe over the study region showed a general single-mode size distribution with the peak mass median aerodynamic diameter (MMAD) at 4.4 µm, shown for 8 individual sets of size-segregated aerosol samples in Fig. 2. Three general size-distribution patterns emerged. Pattern 1: Samples M1, M2, M3, M6, and M7 showed high Fe concentrations in the coarse-mode particles (Fig. 2a). Although M7 was overwhelmingly dominated by coarse-mode particles, this sample did not change the overall size distribution pattern (Fig. 2b). Generally, Fe in the coarse-mode particles accounted for ~68% of the aerosol Fe from all samples and 59% if sample M7 is excluded. This result is consistent with the findings of Maenhaut, *et al*.^45^ who conducted shipboard measurements from August to October between 70°N and 90°N in the region from the Greenland Sea-Fram Strait to the North Pole. Pattern 2: Aerosol Fe in samples M4 and M5 did not vary dramatically between the fine and coarse modes, and Fe in coarse-mode particles accounted for only ~43% of the total Fe loading. Pattern 3: In sample M8, the Fe concentrations on the 0.078 µm stage and 4.4 µm stage were higher than in other samples. Sample M8 was unique in that it was collected along a path adjacent to the Alaskan coastline, where anthropogenic emissions might contribute to aerosol Fe in the fine mode. The size variability of aerosol Fe reflects the changes in atmospheric transport routes over the Arctic Ocean and removal processes along those routes during our study period.

### Dissolvable Fe and fractional Fe solubility

**Figure 3:**
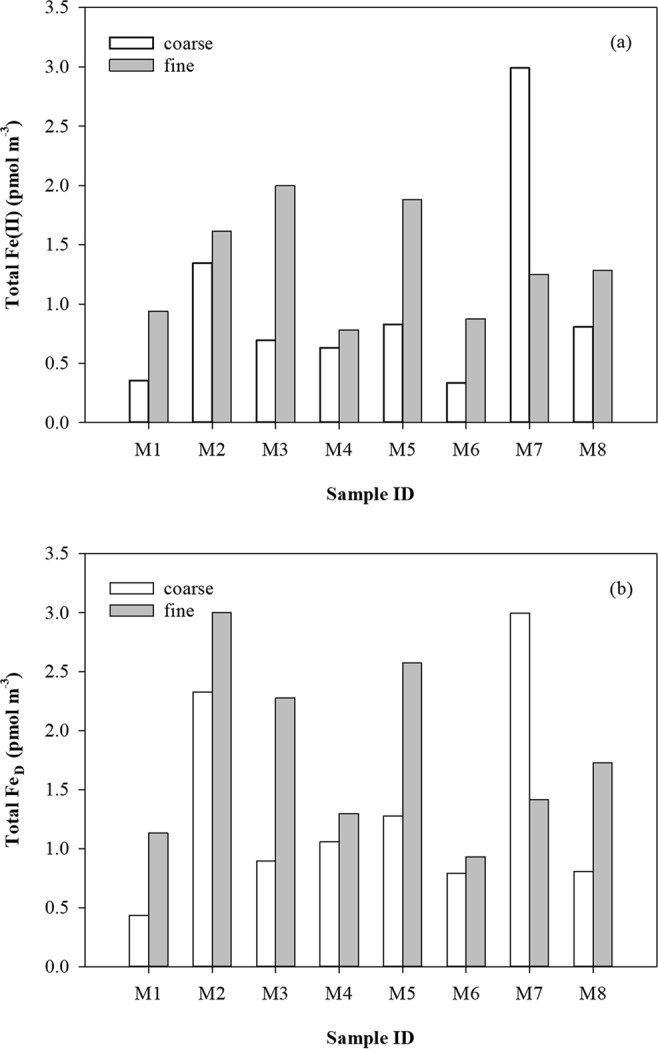
Atmospheric concentrations of dissolvable Fe in fine- and coarse-mode aerosol particles: (**a**) Fe(II); (**b**) Fe_D_.

The concentrations of dissolvable Fe(II) and dissolvable Fe (Fe_D_), defined as the sum of Fe(II) and Fe(III), in fine- and coarse-mode aerosol particles are presented in Fig. 3. The cumulative loading of dissolvable Fe(II) (∑Fe_(II)_) and Fe_D_ (∑Fe_D_) were calculated by the sum of dissolvable Fe(II) and Fe_D_ on each stage in a MOUDI sample, respectively. The concentrations of ∑Fe_(II)_ varied from 1.21 pmol m^−3^ for M6 to 4.24 pmol m^−3^ for M7 (Fig. 3a). Fine-mode Fe(II) ranged from 0.784 pmol m^−3^ to 1.99 pmol m^−3^ with an average of 1.33 (±0.462) pmol m^−3^, accounting for ~61% of ∑Fe_(II)_ across all samples in this region. The concentrations of ∑Fe_D_ ranged from 1.57 pmol m^−3^ for M1 to 5.34 pmol m^−3^ for M2, with an average of 3.12 (±1.33) pmol m^−3^. The concentrations of Fe_D_ in the fine mode ranged from 0.933 pmol m^−3^ to 3.01 pmol m^−3^ (average: 1.79 (±0.745) pmol m^−3^), averaging ~60% of ∑Fe_D_ across all samples (Fig. 3b).

The fractional solubility of aerosol Fe(II) is defined in Equation 1:

1 $$\mathrm{SFe}(\mathrm{II})=100\times \sum \mathrm{Fe}(\mathrm{II})/\sum {{\rm{Fe}}}_{{\rm{T}}}$$

The term SFe(II) is the fractional solubility as a percentage and calculated from the summed concentrations of dissolvable aerosol Fe(II) from each MOUDI (∑Fe(II)) divided by the concurrently collected concentrations of total aerosol Fe (∑Fe_T_).

**Figure 4:**
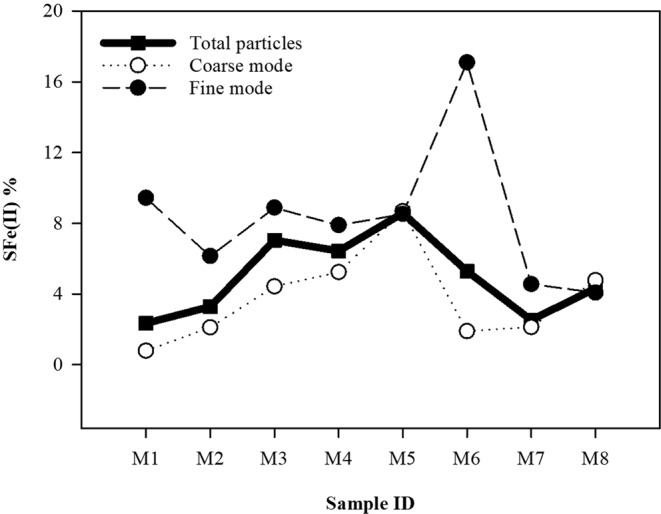
Mean fractional Fe(II) solubility (%) for the fine mode (closed circles), coarse mode (open circles), and total particles (squares).

Aerosol Fe solubility has been determined by various methods, such as using the ratio of Fe(II) to the total Fe in aerosols (SFe(II))^46,47^ and the ratio of total dissolved Fe to the total Fe in aerosols (SFeD)^48,49^, and the results often vary widely. For better comparison with previous results, we also calculated the Fe solubility in the form of SFeD in MOUDI samples and defined it in the same way as for SFe(II) (Table 1). By both measures, the highest values of fractional solubility were found to be associated with samples (M3, M4, M5) collected in areas north of 80°N. The SFe(II) in fine-mode particles ranged from 4.1% to 17% with an average of 8.3 (±4.1) % (Fig. 4). For coarse-mode particles, the range of SFe(II) was from 0.78% to 8.7% (average: 3.8 (±2.6) %), lower than that for fine-mode particles.

**Table 1:** Atmospheric concentrations of dissolvable Fe(II), dissolvable Fe and total aerosol Fe and the fractional solubility for each MOUDI deployment during GN01.

Sample ID	Concentration (pmol m^−3^)			Fractional Fe Solubility (%)	
	∑Fe_(II)_	∑Fe_D_	∑Fe_T_	SFe(II)	SFeD
M1	1.29	1.57	55.1	2.3	2.9
M2	2.96	5.33	90.4	3.3	5.9
M3	2.69	3.17	38.2	7.0	8.3
M4	1.42	2.35	22.0	6.4	11
M5	2.71	3.86	31.6	8.6	12
M6	1.21	1.73	22.8	5.3	7.6
M7	4.24	4.41	168	2.5	2.6
M8	2.10	2.54	48.5	4.3	5.2

### Dry deposition fluxes of aerosol Fe

**Figure 5:**
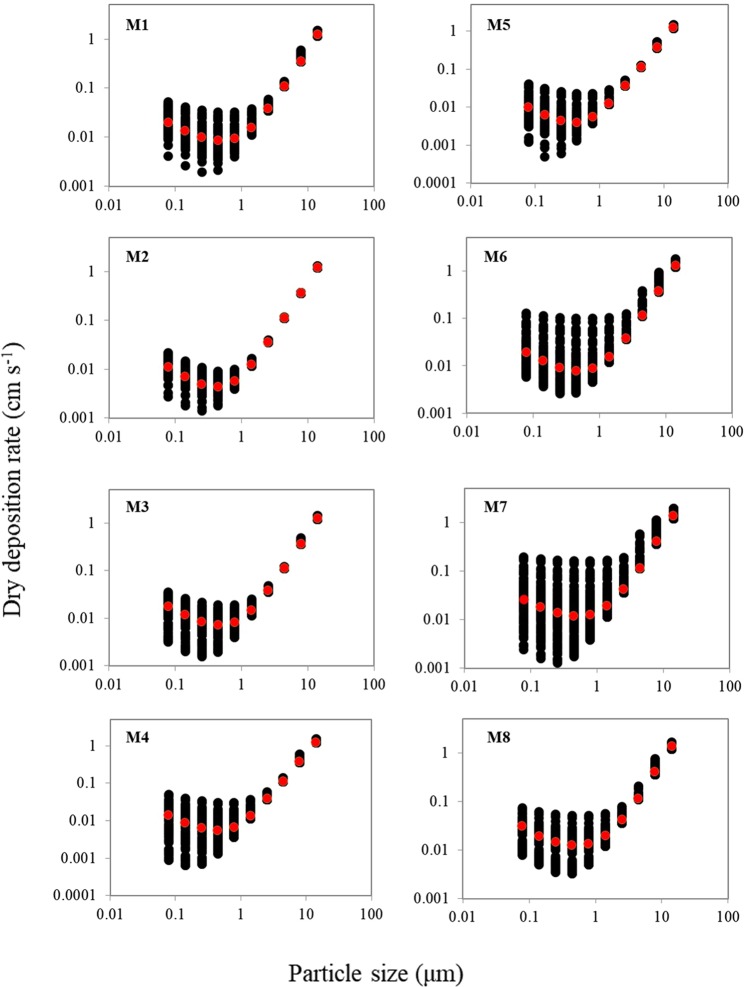
Distributions of dry deposition rates (V_di_) as a function of particle size for each MOUDI sample. Red points are the median values of V_di_ in each size fraction, which was used for calculation of dry deposition flux for that size fraction. Total data points for M1, M2, M3, M4, M5, M6, M7, and M8 were 84, 90, 85, 83, 81, 61, 90 and 65, respectively.

Dry deposition fluxes of aerosol Fe were calculated by applying aerosol Fe concentrations obtained from eight sets of MOUDI samples using Equation 2:

2 $${F}_{d}=\mathop{\sum }\limits_{i=1}^{10}({V}_{di}\times {C}_{Fei})$$

where F_d_ is the dry deposition flux (µmol m^−2^ yr^−1^), C_Fei_ is the concentration of aerosol Fe in each MOUDI stage (µmol m^−3^) applicable to Fe_T_, Fe(II) and Fe_D_, V_di_ is the dry deposition rate for that stage (cm s^−1^). The dry deposition rates were calculated following the combined methods of Slinn and Slinn^50^, Williams^51^, and Quinn and Ondov^52^ which include particle growth at high humidity. The parameter V_di_ is a function of particle size and meteorological conditions (wind speed, air temperature, sea surface temperature, relative humidity (RH)). These parameters, except for RH, were monitored during the cruise and averaged at 2-hour intervals over the 15-second resolution of the original data, while RH was assumed at 80% over the study region based on other studies^53,54^. Figure 5 shows the V_di_ distributions as a function of particle sizes derived from individual MOUDI samples, with the median V_di_ values ranging from less than 0.01 cm s^−1^ to more than 1 cm s^−1^. The variability in dry deposition rates for particle sizes larger than 1μm is less than those for small particles, as V_di_ for large particles is dominated by gravitational settling, while for small particles it is affected mostly by meteorological conditions, such as wind speed.

Table 2 shows the calculated dry deposition fluxes of total aerosol Fe, dissolvable Fe(II) and dissolvable Fe_D_ derived from each MOUDI sample using Eq. 2. These dry deposition fluxes contain substantial uncertainties, on the order of a factor of 2–3 for large particles (>1 μm in diameter) and a factor of 4 for fine particles (0.1–1 μm in diameter), attributed to the uncertainties from V_di_^55^. High **∑**Fe_**T**_ fluxes were found for M1, M2, M7 and M8 collected between 56°N–83°N, ranging from 4.2 µmol m^−2^ yr^−1^ to 6.5 µmol m^−2^ yr^−1^, and the lowest deposition fluxes were associated with samples M3, M4 and M5, north of 82°N, with a range of 0.76 µmol m^−2^ yr^−1^ to 1.8 µmol m^−2^ yr^−1^. High **∑**Fe_**(II)**_ fluxes were associated with M2 and M7 (0.13 µmol m^−2^ yr^−1^), and similar trends existed for **∑**Fe_**D**_ in these samples. Using measurements of the short-lived radioisotope ^7^Be, Marsay, *et al*.^38^ calculated the median bulk (including wet and dry) deposition flux of aerosol Fe along the same cruise transect to be ~18 µmol m^−2^ yr^−1^, which is ~3 times higher than the highest value (6.1 µmol m^−2^ yr^−1^) calculated from MOUDI samples during this study, highlighting an important role for “wet” deposition during the season from late summer to early fall in the Arctic Ocean.

**Table 2:** Dry deposition fluxes of aerosol Fe over the Arctic Ocean (µmol m^−2^ yr^−1^).

Sample ID	∑Fe_(II)_	∑Fe_D_	∑Fe_T_
M1	0.034	0.046	4.2
M2	0.13	0.26	5.6
M3	0.054	0.082	1.8
M4	0.094	0.16	1.6
M5	0.086	0.13	0.76
M6	0.025	0.046	2.6
M7	0.13	0.14	6.0
M8	0.031	0.034	6.5
Average	0.072	0.11	3.6
Std. Dev	0.043	0.076	2.2

## Discussion

### Possible process affecting the depletion of Fe in aerosols over the Arctic

Low ratios of Fe to Ti (a lithogenic tracer^43^) were present in all samples. The Fe/Ti molar ratios from this study ranged from 0.28 to 7.0, with an average of 2.4, which is lower than the typical crustal Fe/Ti ratio of 10 derived from the record of upper continental crust^41^. Similarly low Fe/Ti ratios, relative to the average crustal value, were observed in the bulk aerosol samples collected concurrently during this study^38^ as well as at Alert, Canada by Landsberger *et al*.^42^. While there is speculation that low Fe/Ti ratios may indicate an enrichment of Ti by non-lithogenic Ti sources^43^, the low aerosol Fe/Ti ratios relative to the crustal average observed during this cruise may suggest that average continental crust values are not representative of the source material in this region. The natural variability in source rock mineralogy could be characterized by elemental ratios inherently lower than average crust or could be influenced by processes that favor the removal of Fe. One possible mechanism of Fe depletion at the source is the leaching of Fe from crustal materials. Iron in dust is more soluble than Ti^19^. The leaching process in high latitude surface soil is limited because of the presence of permafrost^56^. However, with increased precipitation driven by local warming^57,58^ and repeated seasonal freeze-thaw cycles, crustal soil materials around the Arctic Ocean may leach out more soluble Fe with time, which could present as an apparent Fe depletion in aerosols derived from these source materials. Pokrovsky, *et al*.^59^ showed that under the influence of multiple freeze-thaw cycles, soluble Fe hydroxide and Fe associated with high-molecular-weight organics might aggregate to transform into colloids, resulting in depletion of the soluble Fe fraction in high Arctic thaw ponds, and this mechanism may also apply to dust sources around the Arctic. Summertime Arctic air is less influenced by long range transport of air masses of continental origin, and thus land areas around the Arctic Ocean that are impacted by freeze-thaw cycles and precipitation could become important summertime sources of aerosol Fe.

### Particle size and potential Fe-organic ligand interactions on Fe solubility

Our data show that relatively high Fe solubility in aerosols was found in the marine atmospheric boundary layer north of 80°N, where polar air masses that have remained over the Arctic Ocean for several days (as determined by AMBT analysis) were sampled. The low mass loading of Fe in the air over that region and the increased importance of smaller particles to aerosol Fe during atmospheric long-range transport could contribute to high Fe solubility, consistent with observations made over other oceanic regions by Baker and Jickells^22^.

Another possible mechanism that may also contribute to the high aerosol Fe solubility in polar air impacted samples is the interactions of aerosol Fe with aerosol organic ligands of marine biogenic origin. Increased solar radiation in the far north during the summer months leads to intensified primary production, and increased concentrations of certain organic acids found in the summertime Arctic marine atmospheric boundary layer have been traced to the sea surface microlayer^60^. Water-soluble carboxylic acid anions (formate, acetate, propionate, and oxalate) measured in the polar air impacted samples accounted for ~10% of the total water-soluble anions analyzed, while the same types of water-soluble anions accounted for only ~2% of the total in samples collected south of 80°N (https://www.bco-dmo.org/dataset/779553; https://www.bco-dmo.org/dataset/779602), which were impacted by mixed marine and continental air masses.

The dissolution of Fe from minerals by organic ligands, including oxalate, has been observed in laboratory studies^61,62^, and field measurements of dust also found positive effects of organic complexation on Fe solubility^63,64^. Thus, enhanced rates of organic emissions during summer in the high latitude Arctic Ocean could influence aerosol Fe fractional solubility. Among three samples collected in the region north of 80°N (M3, M4, M5), relatively good correlations between Fe(II) and oxalate were found for M3 (R^2^ = 0.30) and M5 (R^2^ = 0.47), while there was no such correlation for M4, collected around the North Pole in the presence of shifting sea ice. Although no conclusion can be drawn from these simple correlations between Fe solubility and carboxylic acid anions, we hope that further studies could be designed to address this hypothesis for better understanding of the aerosol Fe-marine ecosystem interactions.

## Methods

### Study region and sampling

This study was carried out as part of the US GEOTRACES Western Arctic GN01 section, with shipboard atmospheric sampling conducted aboard the US Coast Guard Ice Breaker Healy (cruise ID: HLY1502) (Fig. 1). The GN01 section departed from Dutch Harbor, Alaska (53.9°N, 166.5°W) on 9^th^ August 2015, and followed a northward transect through the Bering Strait and across the Makarov Basin to the North Pole. The return to Dutch Harbor followed a southward transect across the Canada Basin and the Bering Sea, and reached port on 12^th^ October 2015.

Aerosol samplers were installed on the forward rail of Healy’s flying bridge, ~23 m above sea level, to minimize the influence of sea spray. To minimize the potential for contamination from the stack exhaust, samplers were forward of the ship’s stack and sampling was controlled by wind speed and direction, through a Campbell Scientific CR800 data-logger interfaced with an anemometer and wind vane set up near the samplers. Aerosol sampling was restricted to periods when in-sector conditions (defined as a relative wind direction from within ±60° of the ship’s bow and a relative wind speed of >0.5 m s^−1^) persisted for at least five continuous minutes. The MOUDI sampler collected size-fractionated aerosols, with 50% cut-off aerodynamic diameters of 0.056, 0.10, 0.18, 0.32, 0.56, 1.0, 1.8, 3.2, 5.6, 10 and 18 µm, on a series of Teflon filters (Pall Corp., 47 mm diameter, 1 µm pore size), with a sampling flowrate of 30 L min^−1^. Both the MOUDI and its pump were housed in enclosures to protect them from rain and sea-spray with an extension tube connected to the MOUDI inlet and extending from the enclosure. A rain shield was installed above the inlet. Due to the anticipated low dust conditions during GN01, and the relatively low frequency of in-sector wind conditions, sample collections lasted for an average of seven days (Table 3).

**Table 3:** Aerosol sampling information along with meteorological parameters from the ship’s underway sensors.

Sample ID	Start date and time	Latitude; Longitude	End date and time	Latitude; Longitude	Sampling volume per filter (m^3^)	AT (°C)	SST (°C)	WS (m/s)
M1	08/10/15 17:53:00	56°04.46N; 170°30.54W	08/17/15 17:15:00	69°55.54N; 167°41.26W	208.8	9.2	8.9	8.4
M2	08/20/15 05:34:00	75°33.99N; 170°44.99W	08/27/15 16:19:00	83°34.33N; 174°43.83E	133.9	−2.9	−1.1	5.0
M3	08/27/15 20:49:00	83°45.46N; 175°02.58E	09/04/15 02:42:00	88°23.96N; 176°38.19E	54.8	−4.2	−1.4	6.3
M4	09/04/15 10:02:00	88°24.53N; 176°45.13E	09/12/15 03:45:00	87°21.09N; 149°25.82W	119.2	−5.1	−1.4	5.5
M5	09/12/15 06:13:00	87°16.25N; 149°02.66W	09/20/15 22:13:00	82°15.52N; 149°22.63W	53.5	−9.5	−4.1	4.3
M6	09/21/15 01:10:00	82°06.03N; 150°48.66W	09/26/15 03:05:00	78°58.42N; 148°30.06W	99.0	−4.3	−1.4	7.8
M7	09/26/15 04:38:00	78°48.21N; 148°05.59W	10/03/15 16:25:00	73°25.56N; 156°47.59W	102.8	−2.2	−1.1	8.7
M8	10/03/15 18:41:00	73°23.83N; 156°45.95W	10/09/15 23:47:00	65°57.02N; 168°26.91W	74.3	−2.2	0.8	9.6

All times are in UTC. AT is atmospheric temperature, SST is sea surface temperature, WS is wind speed, and the mean values of these parameters for each sampling period are reported.

Clean polyethylene gloves were worn for loading and unloading of sample filters, which were carried out underneath a high-efficiency particulate air (HEPA) filter blower within a plastic “bubble” clean area constructed in the ship’s main laboratory. Filters were loaded onto each stage of the MOUDI impactor from labeled petri dishes using pre-cleaned Teflon tweezers and were transferred back to the same petri dishes after sample recovery. Filter holders were double-bagged for transfer between the ship’s laboratory and the samplers. Deployment blanks were carried out using the same protocols, but with the pumps turned off. All sample and field blank filters were subsequently double-bagged and stored frozen until analysis.

### Sample analyses

#### Total Fe in aerosols

Aerosol samples were analyzed for the total concentrations of atmospheric Fe and Ti by a sector field inductively coupled plasma-mass spectrometer (SF-ICPMS) in the Rutgers Inorganic Analytical Laboratory at the Department of Marine Sciences of Rutgers University, following a previously described digestion protocol^47^. Briefly, a portion of each sample filter was placed in a 15 mL Teflon vial with a mixture of concentrated HNO_3_ (0.8 ml) and HF (0.1 ml) (Optima, Fisher Sci.) and digested for 4 hours on a hot plate at 160 °C. Each digestion solution was evaporated to dryness, followed by the addition of 2 ml 0.5M HNO_3_ and 1 ppb Indium (In) for ICP-MS drift correction. Both field blanks and procedure blanks were treated in the same way as samples. All Teflon vials were acid-cleaned, and all procedures were carried out in a class-100 clean-room hood in the lab. The sample digestion procedures were assessed using Standard Reference Material (SRM) 1648a (National Institute of Standards and Technology, NIST, Gaithersburg, MD), subsamples of which were treated under the same conditions as for samples. The digest recoveries based on SRM1648a ranged between 89–99% for Fe and 83–94% for Ti (n = 7) which were close enough to the measured quantities that no yield correction needed to be applied, and the precision determined from sample splits and duplicate digest aliquots ranged between 93–106% for Fe and between 85–124% for Ti (n = 10). The method detection limits were 0.691 pmol m^−3^ for Fe and 2.58 pmol m^−3^ for Ti, which were obtained based on three times the standard deviation of a total of 14 filter blanks and a nominal 100 m^3^ sampling volume. A series of external calibration standards were run at the beginning and then at the end of the analyses. More details on the ICP-MS instrument settings can be found in Annett, *et al*.^65^.

#### Dissolvable Fe in aerosols

The concentrations of dissolvable Fe(II) and Fe(III) in aerosol samples were obtained using UV/Visible spectroscopy with a modified Ferrozine method^47^. The leaching solution for samples was 0.5 mM ammonium acetate that was filtered through a Nuclepore track-etch membrane filter (47 mm, 0.2 μm) and adjusted to ~pH 5.3. The leaching conditions were chosen to simulate cloud water conditions for marine aerosols, similar to previous studies that used formate-, acetate-, and ammonium acetate-based buffer solutions for simulating precipitation conditions^46,64,66^. A brief description of the procedures is as follows: a portion of each sample filter was first placed into a leaching solution of ammonium acetate (0.5 mM) for 1 h, and then the leachate was split into two parts, one for Fe(II) determination and the other for total dissolvable Fe. A solution of 0.01 M hydroxylamine hydrochloride solution (1%) was added to the total dissolvable Fe filtrate portion to reduce Fe(III) to Fe(II), and the sample solution was set aside for 1 h to ensure complete reduction before adding the same ferrozine solution as for the Fe(II) filtrate portion. The Fe(II) measured in this way was considered as total dissolvable Fe. After these procedures, each sample leaching solution was filtered through a 13 mm polytetrafluorethylene syringe filter of 0.2 μm pore size. All field blanks were treated in the same way as samples. The concentrations of Fe(II) in sample solutions were determined at 562 nm using a TIDAS-1 spectrometer module with a 200 cm liquid waveguide capillary flow cell (World Precision Instruments Inc., FL, USA). The detection limit of the method for Fe(II) was 0.30 nM, calculated as three times the standard deviation of the measured blank values (n = 5).

### Air mass back-trajectory analyses

The NOAA HYSPLIT4 model was used to calculate normal and ensemble air mass back-trajectories (AMBT) based on Stein, *et al*.^67^ and Rolph, *et al*.^68^ (Mukherjee, P. *et al*., manuscript in preparation). Briefly, with the ship’s location where each sample collection began as the model starting point, a four-day back-trajectory was calculated at six-hour intervals along the cruise track and then a total of 244 normal trajectories (4 trajectories/day × 61 days) were clustered along with the percentage of trajectories in each cluster.

## Data Availability

The data presented in this paper are available in the websites: https://www.bco-dmo.org/dataset/779688; https://www.bco-dmo.org/dataset/779649.
